# *N*-(2,4-Dimethyl­phen­yl)succinimide

**DOI:** 10.1107/S1600536810009694

**Published:** 2010-03-20

**Authors:** B. S. Saraswathi, B. Thimme Gowda, Sabine Foro, Hartmut Fuess

**Affiliations:** a Department of Chemistry, Mangalore University, Mangalagangotri 574 199, Mangalore, India; b Institute of Materials Science, Darmstadt University of Technology, Petersenstrasse 23, D-64287 Darmstadt, Germany

## Abstract

In the title compound, C_12_H_13_NO_2_, the dihedral angle between the benzene ring and the imide segment is 85.7 (1)°. In the crystal, the mol­ecules are packed into zigzag chains parallel to the *a* axis.

## Related literature

For our study of the effect of ring and side-chain substitutions on the structures of biologically significant compounds, see: Gowda *et al.* (2007 ▶); Saraswathi *et al.* (2010**a* ▶,b* ▶).
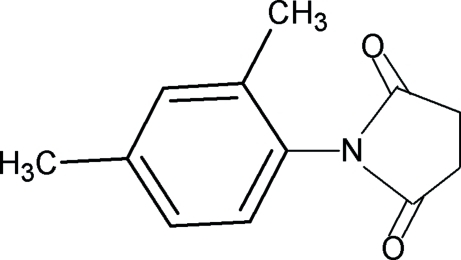


## Experimental

### 

#### Crystal data

C_12_H_13_NO_2_
*M* *_r_* = 203.23
Orthorhombic, 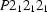

*a* = 7.1461 (7) Å
*b* = 11.182 (2) Å
*c* = 13.676 (2) Å
*V* = 1092.8 (3) Å^3^
*Z* = 4
Cu *K*α radiation
μ = 0.68 mm^−1^
*T* = 299 K
0.50 × 0.25 × 0.25 mm

#### Data collection

Enraf–Nonius CAD-4 diffractometer
2947 measured reflections
1152 independent reflections
987 reflections with *I* > 2σ(*I*)
*R*_int_ = 0.065
3 standard reflections every 120 min  intensity decay: 1.0%

#### Refinement

*R*[*F* ^2^ > 2σ(*F* ^2^)] = 0.041
*wR*(*F* ^2^) = 0.113
*S* = 1.06
1152 reflections
157 parameters
H atoms treated by a mixture of independent and constrained refinement
Δρ_max_ = 0.12 e Å^−3^
Δρ_min_ = −0.17 e Å^−3^

### 

Data collection: *CAD-4-PC* (Enraf–Nonius, 1996 ▶); cell refinement: *CAD-4-PC*; data reduction: *REDU4* (Stoe & Cie, 1987 ▶); program(s) used to solve structure: *SHELXS97* (Sheldrick, 2008 ▶); program(s) used to refine structure: *SHELXL97* (Sheldrick, 2008 ▶); molecular graphics: *PLATON* (Spek, 2009 ▶); software used to prepare material for publication: *SHELXL97*.

## Supplementary Material

Crystal structure: contains datablocks I, global. DOI: 10.1107/S1600536810009694/vm2021sup1.cif

Structure factors: contains datablocks I. DOI: 10.1107/S1600536810009694/vm2021Isup2.hkl

Additional supplementary materials:  crystallographic information; 3D view; checkCIF report

## Figures and Tables

**Fig. 1.**
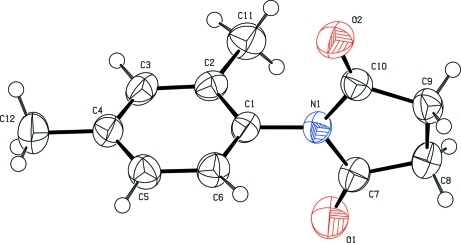
Molecular structure of the title compound, showing the atom labelling scheme. The displacement ellipsoids are drawn at the 50% probability level. The H atoms are represented as small spheres of arbitrary radii.

**Fig. 2.**
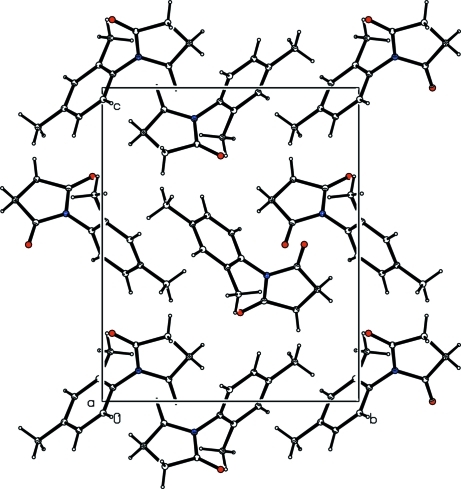
Molecular packing of the title compound.

## supplementary crystallographic information

### Comment

As a part of studying the effect of ring and side chain substitutions on the structures of biologically significant compounds (Gowda *et al.*, 2007; Saraswathi *et al.*, 2010*a,b*), the crystal structure of *N*,*N*-(2,4-dimethylphenyl)succinimide has been determined (Fig. 1). The dihedral angle between the benzene ring and the imide segment in the molecule is 85.7 (1)°.

The torsional angles of the groups, C2 - C1 - N1 - C7, C6 - C1 - N1 - C7, C2 - C1 - N1 - C10 and C6 - C1 - N1 - C10 in the molecule are -97.8 (3)°, 80.0 (3)°, 88.9 (3)° and -93.4 (3)°, respectively, while the torsional angles of the groups, O1 - C7 - N1 - C1, C8 - C7 - N1 - C1, O2 - C10 - N1 - C1 and C9 - C10 - N1 - C1 are 3.2 (4)°, -178.6 (2)°, -7.2 (4)° and 173.0 (2)°, respectively.

The packing of molecules into zigzag chains is shown in Fig.2.

### Experimental

The solution of succinic anhydride (0.025 mole) in toluene (25 ml) was treated dropwise with the solution of 2,4-dimethylaniline (0.025 mole) also in toluene (20 ml) with constant stirring. The resulting mixture was stirred for one h and set aside for an additional hour at room temperature for the completion of reaction. The mixture was then treated with dilute hydrochloric acid to remove the unreacted 2,4-dimethylaniline. The resultant solid *N*-(2,4-dimethylphenyl)succinamic acid was filtered under suction and washed thoroughly with water to remove the unreacted succinic anhydride and succinic acid. It was recrystallized to constant melting point from ethanol.

*N*-(2,4-Dimethylphenyl)succinamic acid was heated for 2 h and then allowed to cool slowly to room temperature to get the compound, *N*-(2,4-dimethylphenyl)succinimide. The purity of the compound was checked and characterized by its infrared spectra.

The rod like colourless single crystals of the compound used in X-ray diffraction studies were grown in ethanolic solution by a slow evaporation at room temperature.

### Refinement

The H atoms of the CH_3_ groups were positioned with idealized geometry using a riding model with C—H = 0.96 Å. The other H atoms were located in a difference map and their position refined to C—H = 0.91 (3)–1.06 (3) Å. All H atoms were refined with isotropic displacement parameters (set to 1.2 times of the *U*_eq_ of the parent atom).

In the absence of significant anomalous dispersion effects, Friedel pairs were merged and the Δf"term set to zero.

### Crystal data

**Table:**

C_12_H_13_NO_2_	*F*(000) = 432
*M**_r_* = 203.23	*D*_x_ = 1.235 Mg m^−^^3^
Orthorhombic, *P*2_1_2_1_2_1_	Cu *K*α radiation, λ = 1.54180 Å
Hall symbol: P 2ac 2ab	Cell parameters from 25 reflections
*a* = 7.1461 (7) Å	θ = 5.1–23.4°
*b* = 11.182 (2) Å	µ = 0.68 mm^−^^1^
*c* = 13.676 (2) Å	*T* = 299 K
*V* = 1092.8 (3) Å^3^	Rod, colourless
*Z* = 4	0.50 × 0.25 × 0.25 mm

### Data collection

**Table:**

Enraf–Nonius CAD-4 diffractometer	*R*_int_ = 0.065
Radiation source: fine-focus sealed tube	θ_max_ = 66.9°, θ_min_ = 5.1°
graphite	*h* = −3→8
ω/2θ scans	*k* = −13→13
2947 measured reflections	*l* = 0→16
1152 independent reflections	3 standard reflections every 120 min
987 reflections with *I* > 2σ(*I*)	intensity decay: 1.0%

### Refinement

**Table:**

Refinement on *F*^2^	Primary atom site location: structure-invariant direct methods
Least-squares matrix: full	Secondary atom site location: difference Fourier map
*R*[*F*^2^ > 2σ(*F*^2^)] = 0.041	Hydrogen site location: inferred from neighbouring sites
*wR*(*F*^2^) = 0.113	H atoms treated by a mixture of independent and constrained refinement
*S* = 1.06	*w* = 1/[σ^2^(*F*_o_^2^) + (0.0693*P*)^2^ + 0.082*P*] where *P* = (*F*_o_^2^ + 2*F*_c_^2^)/3
1152 reflections	(Δ/σ)_max_ = 0.011
157 parameters	Δρ_max_ = 0.12 e Å^−^^3^
0 restraints	Δρ_min_ = −0.17 e Å^−^^3^

### Special details

**Table:**

Geometry. All esds (except the esd in the dihedral angle between two l.s. planes) are estimated using the full covariance matrix. The cell esds are taken into account individually in the estimation of esds in distances, angles and torsion angles; correlations between esds in cell parameters are only used when they are defined by crystal symmetry. An approximate (isotropic) treatment of cell esds is used for estimating esds involving l.s. planes.
Refinement. Refinement of *F*^2^ against ALL reflections. The weighted *R*-factor wR and goodness of fit *S* are based on *F*^2^, conventional *R*-factors *R* are based on *F*, with *F* set to zero for negative *F*^2^. The threshold expression of *F*^2^ > σ(*F*^2^) is used only for calculating *R*-factors(gt) etc. and is not relevant to the choice of reflections for refinement. *R*-factors based on *F*^2^ are statistically about twice as large as those based on *F*, and *R*- factors based on ALL data will be even larger.

### Fractional atomic coordinates and isotropic or equivalent isotropic displacement parameters (Å^2^)

**Table:**

	*x*	*y*	*z*	*U*_iso_*/*U*_eq_	
C1	−0.0480 (4)	0.0411 (2)	0.04393 (17)	0.0377 (6)	
C2	−0.2151 (4)	−0.0128 (2)	0.07236 (18)	0.0410 (6)	
C3	−0.2763 (4)	−0.1082 (2)	0.0153 (2)	0.0461 (6)	
H3	−0.400 (5)	−0.144 (3)	0.034 (2)	0.055*	
C4	−0.1827 (4)	−0.1484 (2)	−0.06581 (18)	0.0469 (6)	
C5	−0.0181 (4)	−0.0909 (3)	−0.0921 (2)	0.0488 (7)	
H5	0.042 (5)	−0.111 (3)	−0.153 (2)	0.059*	
C6	0.0488 (4)	0.0035 (3)	−0.0372 (2)	0.0462 (7)	
H6	0.184 (5)	0.035 (3)	−0.049 (2)	0.055*	
C7	−0.0064 (4)	0.2593 (2)	0.06994 (18)	0.0429 (6)	
C8	0.0891 (5)	0.3392 (2)	0.1425 (2)	0.0483 (7)	
H8A	0.164 (5)	0.390 (3)	0.107 (2)	0.058*	
H8B	−0.013 (5)	0.391 (3)	0.1798 (19)	0.058*	
C9	0.1992 (5)	0.2553 (3)	0.2077 (2)	0.0478 (7)	
H9A	0.182 (5)	0.264 (3)	0.273 (3)	0.057*	
H9B	0.325 (5)	0.267 (3)	0.193 (2)	0.057*	
C10	0.1393 (4)	0.1310 (2)	0.17959 (17)	0.0427 (6)	
C11	−0.3240 (5)	0.0311 (3)	0.1583 (2)	0.0581 (8)	
H11A	−0.3491	0.1150	0.1507	0.070*	
H11B	−0.2529	0.0185	0.2169	0.070*	
H11C	−0.4400	−0.0118	0.1625	0.070*	
C12	−0.2569 (6)	−0.2502 (3)	−0.1256 (2)	0.0683 (10)	
H12A	−0.2704	−0.3197	−0.0849	0.082*	
H12B	−0.1714	−0.2674	−0.1779	0.082*	
H12C	−0.3765	−0.2288	−0.1523	0.082*	
N1	0.0217 (3)	0.14153 (17)	0.09856 (13)	0.0376 (5)	
O1	−0.0975 (3)	0.28747 (18)	−0.00034 (16)	0.0666 (7)	
O2	0.1797 (4)	0.03828 (17)	0.21710 (15)	0.0634 (6)	

### Atomic displacement parameters (Å^2^)

**Table:**

	*U*^11^	*U*^22^	*U*^33^	*U*^12^	*U*^13^	*U*^23^
C1	0.0370 (13)	0.0333 (12)	0.0428 (12)	−0.0018 (10)	−0.0027 (11)	0.0014 (10)
C2	0.0362 (13)	0.0422 (12)	0.0447 (12)	−0.0015 (11)	0.0014 (12)	0.0064 (10)
C3	0.0418 (14)	0.0410 (13)	0.0554 (14)	−0.0095 (12)	−0.0025 (13)	0.0064 (11)
C4	0.0557 (16)	0.0388 (12)	0.0462 (13)	−0.0038 (13)	−0.0092 (13)	0.0017 (11)
C5	0.0505 (15)	0.0511 (15)	0.0448 (13)	0.0000 (13)	0.0040 (14)	−0.0067 (12)
C6	0.0380 (14)	0.0486 (15)	0.0521 (14)	−0.0044 (12)	0.0055 (13)	−0.0029 (11)
C7	0.0385 (13)	0.0368 (12)	0.0534 (13)	0.0039 (11)	−0.0018 (13)	−0.0007 (11)
C8	0.0501 (16)	0.0375 (13)	0.0571 (15)	−0.0026 (13)	−0.0003 (15)	−0.0054 (12)
C9	0.0444 (15)	0.0526 (15)	0.0463 (14)	−0.0084 (14)	−0.0032 (14)	−0.0062 (12)
C10	0.0390 (13)	0.0472 (14)	0.0419 (12)	−0.0021 (12)	−0.0006 (11)	−0.0001 (11)
C11	0.0522 (17)	0.0627 (17)	0.0595 (16)	−0.0011 (16)	0.0141 (15)	−0.0013 (14)
C12	0.083 (3)	0.0562 (17)	0.0657 (17)	−0.0156 (18)	−0.0154 (19)	−0.0090 (15)
N1	0.0353 (11)	0.0353 (10)	0.0422 (10)	−0.0008 (9)	−0.0031 (9)	−0.0015 (8)
O1	0.0745 (15)	0.0501 (12)	0.0754 (12)	0.0086 (11)	−0.0306 (13)	0.0057 (11)
O2	0.0710 (15)	0.0525 (12)	0.0665 (12)	−0.0038 (11)	−0.0214 (12)	0.0138 (10)

### Geometric parameters (Å, °)

**Table:**

C1—C6	1.373 (4)	C8—C9	1.514 (4)
C1—C2	1.393 (4)	C8—H8A	0.92 (3)
C1—N1	1.438 (3)	C8—H8B	1.06 (3)
C2—C3	1.392 (4)	C9—C10	1.505 (4)
C2—C11	1.493 (4)	C9—H9A	0.91 (3)
C3—C4	1.372 (4)	C9—H9B	0.93 (4)
C3—H3	1.00 (3)	C10—O2	1.192 (3)
C4—C5	1.388 (4)	C10—N1	1.396 (3)
C4—C12	1.498 (4)	C11—H11A	0.9600
C5—C6	1.381 (4)	C11—H11B	0.9600
C5—H5	0.97 (3)	C11—H11C	0.9600
C6—H6	1.04 (3)	C12—H12A	0.9600
C7—O1	1.203 (3)	C12—H12B	0.9600
C7—N1	1.388 (3)	C12—H12C	0.9600
C7—C8	1.499 (4)		
			
C6—C1—C2	121.6 (2)	H8A—C8—H8B	108 (3)
C6—C1—N1	119.0 (2)	C10—C9—C8	105.9 (2)
C2—C1—N1	119.3 (2)	C10—C9—H9A	108 (2)
C1—C2—C3	116.4 (2)	C8—C9—H9A	116 (2)
C1—C2—C11	121.6 (2)	C10—C9—H9B	110 (2)
C3—C2—C11	122.0 (2)	C8—C9—H9B	106.8 (19)
C4—C3—C2	123.5 (3)	H9A—C9—H9B	109 (3)
C4—C3—H3	120.1 (17)	O2—C10—N1	124.1 (2)
C2—C3—H3	116.3 (17)	O2—C10—C9	128.7 (2)
C3—C4—C5	118.1 (2)	N1—C10—C9	107.2 (2)
C3—C4—C12	121.2 (3)	C2—C11—H11A	109.5
C5—C4—C12	120.7 (3)	C2—C11—H11B	109.5
C6—C5—C4	120.4 (3)	H11A—C11—H11B	109.5
C6—C5—H5	120 (2)	C2—C11—H11C	109.5
C4—C5—H5	120 (2)	H11A—C11—H11C	109.5
C1—C6—C5	120.0 (3)	H11B—C11—H11C	109.5
C1—C6—H6	119.3 (16)	C4—C12—H12A	109.5
C5—C6—H6	119.7 (16)	C4—C12—H12B	109.5
O1—C7—N1	123.5 (2)	H12A—C12—H12B	109.5
O1—C7—C8	128.3 (2)	C4—C12—H12C	109.5
N1—C7—C8	108.2 (2)	H12A—C12—H12C	109.5
C7—C8—C9	104.9 (2)	H12B—C12—H12C	109.5
C7—C8—H8A	106.3 (19)	C7—N1—C10	113.0 (2)
C9—C8—H8A	113 (2)	C7—N1—C1	122.9 (2)
C7—C8—H8B	109.2 (18)	C10—N1—C1	123.7 (2)
C9—C8—H8B	114.2 (15)		
			
C6—C1—C2—C3	1.2 (4)	C7—C8—C9—C10	−8.4 (3)
N1—C1—C2—C3	178.9 (2)	C8—C9—C10—O2	−173.7 (3)
C6—C1—C2—C11	−177.6 (2)	C8—C9—C10—N1	6.0 (3)
N1—C1—C2—C11	0.0 (4)	O1—C7—N1—C10	177.2 (3)
C1—C2—C3—C4	−0.9 (4)	C8—C7—N1—C10	−4.6 (3)
C11—C2—C3—C4	178.0 (3)	O1—C7—N1—C1	3.2 (4)
C2—C3—C4—C5	0.1 (4)	C8—C7—N1—C1	−178.6 (2)
C2—C3—C4—C12	−178.9 (3)	O2—C10—N1—C7	178.8 (3)
C3—C4—C5—C6	0.5 (4)	C9—C10—N1—C7	−0.9 (3)
C12—C4—C5—C6	179.5 (3)	O2—C10—N1—C1	−7.2 (4)
C2—C1—C6—C5	−0.7 (4)	C9—C10—N1—C1	173.0 (2)
N1—C1—C6—C5	−178.4 (3)	C6—C1—N1—C7	80.0 (3)
C4—C5—C6—C1	−0.2 (4)	C2—C1—N1—C7	−97.8 (3)
O1—C7—C8—C9	−173.9 (3)	C6—C1—N1—C10	−93.4 (3)
N1—C7—C8—C9	8.1 (3)	C2—C1—N1—C10	88.9 (3)
